# SHARED SPATIAL EFFECTS ON QUANTITATIVE GENETIC PARAMETERS: ACCOUNTING FOR SPATIAL AUTOCORRELATION AND HOME RANGE OVERLAP REDUCES ESTIMATES OF HERITABILITY IN WILD RED DEER

**DOI:** 10.1111/j.1558-5646.2012.01620.x

**Published:** 2012-08

**Authors:** Katie V Stopher, Craig A Walling, Alison Morris, Fiona E Guinness, Tim H Clutton-Brock, Josephine M Pemberton, Daniel H Nussey

**Affiliations:** 1 Institute of Evolutionary Biology, University of EdinburghEdinburgh, EH9 3JT, United Kingdom; 3 Department of Zoology, University of CambridgeCambridge, CB2 3EJ, United Kingdom; 4 Centre for Immunity, Infection and Evolution, University of EdinburghAshworth Labs, West Mains Road, Edinburgh EH9 3JT, United Kingdom

**Keywords:** Additive genetic variance, “animal model, ” maternal effects, microevolution, resource heterogeneity

## Abstract

Social structure, limited dispersal, and spatial heterogeneity in resources are ubiquitous in wild vertebrate populations. As a result, relatives share environments as well as genes, and environmental and genetic sources of similarity between individuals are potentially confounded. Quantitative genetic studies in the wild therefore typically account for easily captured shared environmental effects (e.g., parent, nest, or region). Fine-scale spatial effects are likely to be just as important in wild vertebrates, but have been largely ignored. We used data from wild red deer to build “animal models” to estimate additive genetic variance and heritability in four female traits (spring and rut home range size, offspring birth weight, and lifetime breeding success). We then, separately, incorporated spatial autocorrelation and a matrix of home range overlap into these models to estimate the effect of location or shared habitat on phenotypic variation. These terms explained a substantial amount of variation in all traits and their inclusion resulted in reductions in heritability estimates, up to an order of magnitude up for home range size. Our results highlight the potential of multiple covariance matrices to dissect environmental, social, and genetic contributions to phenotypic variation, and the importance of considering fine-scale spatial processes in quantitative genetic studies.

Additive genetic variance (*V*_A_) and heritability (*h*^2^, the ratio of genetic to phenotypic variance) are fundamental parameters in our understanding of the evolutionary potential and dynamics of traits in nature (Lande 1982; Houle 1992). Quantitative genetic models rely on the phenotypic similarities between relatives to estimate them (Falconer and Mackay 1996; Lynch and Walsh 1998). The application of “animal models,” a form of mixed-effects model in which *V*_A_ is estimated using a genetic relatedness matrix derived from a multi-generational pedigree (Lynch and Walsh 1998), in wild populations has advanced our understanding of evolutionary genetics in nature (Ellegren and Sheldon 2008; Kruuk et al. 2008). However, wild populations are characterized by high levels of environmental heterogeneity and relatives often share environments. It has been argued that the multi-generational approach of the “animal model” to estimating heritability reduces bias from environmental similarities because the model uses both phenotypic resemblance between close relatives and more distant relatives, who are less likely to live under similar environmental conditions (Postma and Charmantier 2007). Nonetheless, failing to properly account for such shared environmental effects is known to bias estimates of parameters derived from “animal models” (Kruuk and Hadfield 2007), and it has become common practice to account for certain kinds of shared environmental effects (e.g., parental identity, nest, group, or region of study area) by incorporating these into models as fixed or random effects (e.g., Kruuk et al. 2001; MacColl and Hatchwell 2003; Charmantier et al. 2004; Wilson et al. 2005; Kruuk and Hadfield 2007).

However, beyond these shared environment effects, social structure and natal philopatry—both of which are ubiquitous in wild vertebrates—are likely to result in spatial associations among relatives throughout individuals’ lives. Where relatives are associated in space throughout their lives, and the environment is spatially heterogeneous, it follows that relatives are more likely to experience similar fine-scale environmental effects than nonrelatives. Relatives will therefore show greater resemblance to one another. If related individuals share both genes and space, the potential exists for a positive correlation between genetic relatedness and similarity resulting from spatial effects. Although more challenging to incorporate within “animal models” than most shared environments currently considered in the wild animal literature, like all nongenetic causes of phenotypic similarity between relatives spatial similarities have clear potential to bias estimates of *V*_A_ and *h*^2^, as well as other components of variance (Falconer and Mackay 1996). To date, the importance of spatial similarity in quantitative genetic studies of wild vertebrates has been largely dismissed. Here, we examine the effects of spatial autocorrelation (SAC) and home range overlap on phenotypic variation and their potential to bias estimates of *V*_A_ and *h*^2^ in a wild red deer population.

SAC is the dependence of a given variable's value on the values of the same variable measured at nearby locations (Cliff and Ord 1981; Fortin and Dale 2005). It has long been recognized as a source of bias in quantitative genetic analyses of plant agriculture and forestry studies (Cullis and Gleeson 1989, 1991; Burgueno et al. 2000; Costa e Silva et al. 2001), as well as more generally in ecology, both as a source of bias but also in identification of relevant and interesting spatial processes (Legendre 1993; Kissling and Carl 2008; Fortin and Dale 2009). In quantitative genetic analyses of agricultural and forestry trials, SAC can be accounted for to some extent by experimental design and appropriate fitting of block effects. However, particularly in forestry trials, substantial heterogeneity may exist within sites that can be further modeled by the inclusion of particular SAC functions (Dutkowski et al. 2002). Simulation studies have shown that variance component estimates in mixed-effects models were upwardly biased when positive SAC was not accounted for (Magnussen 1993), although other forestry studies have found that accounting for SAC can have differing effects on estimates of additive genetic variation (Costa e Silva et al. 2001; Dutkowski et al. 2002).

In studies of wild animals, the effect of SAC on estimates of quantitative genetic parameters has received little attention. The notable exception is a study of laying date and clutch size in a wild great tit population, which used parent–offspring regression to estimate *V*_A_ and *h*^2^ (van der Jeugd and McCleery 2002). Here, it was found that failure to account for SAC resulted in substantial overestimation (more than 60%) of heritability in laying date, but not in clutch size. Although suggesting that SAC can in some cases represent an important source of both phenotypic variation and bias in quantitative genetic analyses, this study did not apply particularly powerful or informative statistical techniques. Parent–offspring regression conflates parental environment and genetic effects; the “animal model” provides a much more powerful tool for accurately estimating *V*_A_ and separating environmental and genetic sources of variance (Lynch and Walsh 1998; Kruuk 2004). Furthermore, the study examined SAC effects by simply comparing parent–offspring regressions among groups of parents and offspring breeding at three different distances apart (van der Jeugd and McCleery 2002). In fact, as the forestry studies discussed above illustrate, autocorrelation functions can be simultaneously estimated and accounted for directly within mixed-effects models that also estimate *V*_A_ and from which *h*^2^ can therefore be calculated. To our knowledge, such an approach has yet to be applied to test the importance or nature of SAC underlying phenotypic variation, or its effects on parameter estimates from “animal models,” in any wild vertebrate system.

Implementation of SAC functions within mixed models requires individuals to be assigned specific spatial locations (e.g., average lifetime locations, locations of nest). However, most animals are mobile and home range sizes and shapes are likely to vary markedly between individuals. Methods for specifically assessing the importance of home range overlap effects on phenotypic variation and in estimating quantitative genetic parameters are therefore also desirable. In an “animal model,” a matrix of pairwise genetic relatedness coefficients (the “A matrix”) among individuals in a population is fitted within a mixed-effects model to estimate *V*_A_ as the phenotypic similarity among relatives (Henderson 1953, 1976). In animal breeding, it is relatively common practice to fit additional matrices to estimate dominance or epistatic genetic variance (e.g., Smith and Maki-Tanila 1990; Palucci et al. 2007). Multi-matrix approaches (fitting additional vectors of random effects with their associated covariance matrices) have recently been advocated to estimate and account for shared environment effects alongside genetic effects (Danchin et al. 2011), but have yet to be implemented empirically. Coefficients measuring the degree of home range overlap among individuals, or indeed any measure of social or spatial association, can readily be calculated if sufficient spatial or social interaction data are available. Dyadic home range overlap coefficients, assuming they scale similarly to the relatedness coefficients in the A matrix, can be built into a matrix incorporating all pairwise comparisons among individuals (which we term the “S matrix”) and fitted as a random effect alongside the A matrix (see methods). Such a “double matrix” approach would yield estimates of both *V*_A_ and the variance attributable to home range overlap among individuals and could provide important insight into shared environmental effects and reduce bias in estimates of heritability.

## THE PRESENT STUDY

In this study, we test the importance of shared environmental effects on four female phenotypic traits—rut home range size (RHR), spring home range size (SHR), offspring birth weight (BW), and lifetime breeding success (LBS)—in a wild population of red deer. In this species, females are strongly philopatric, with little dispersal from their natal sites, and the majority of females associate in loosely matrilineal groups (Clutton-Brock et al. 1982). As a result, we observe fine-scale genetic structure within the female population (Nussey et al. 2005c). Habitat and vegetation types are also highly spatially structured within the study area (Clutton-Brock et al. 1982; McLoughlin et al. 2006), so relatives may have similar phenotypes due to shared adult environment rather than genes. We chose to investigate variation in home range sizes as focal traits because they are by definition likely to be spatially autocorrelated due to their dependency on food availability. Females are expected to trade-off the range size needed to acquire sufficient food with the energy required to move across this range (McNab 1963); therefore, home range size will be dependent upon the availability and quality of forage and so is expected to vary spatially over the study area. Consequently, we predicted large shared environment effects and considerable bias in estimates of heritability in these two home range size measures. Offspring BW is a heritable but also highly plastic maternal trait that correlates with female annual reproductive performance in this population (Coulson et al. 2003; Nussey et al. 2005a; Stopher et al. 2008), although the importance of shared environmental effects during adulthood on this trait has yet to be determined. Finally, lifetime breeding success is a trait of significant evolutionary importance as a measure of individual fitness across many taxa (Merila and Sheldon 2000; Rodriguez-Munoz et al. 2010). It has been shown not to be heritable but to be associated with resource selection by females in this population (Kruuk et al. 2000; McLoughlin et al. 2006). Developing our understanding of the fine-scale environmental causes of variation in such fitness-related traits is central to understanding evolutionary dynamics in natural systems.

Previous quantitative genetic studies of this population have used “animal models” to estimate *V*_A_ and calculate *h*^2^ in various traits, while also illustrating the importance of simultaneously accounting for nongenetic among individual variation (so-called “permanent environment” effects), maternal and matrilineal effects (Kruuk et al. 2000; Kruuk and Hadfield 2007). Here, we extend such models in our four selected traits by fitting spatial information using two different but not mutually incompatible techniques: (1) incorporating SAC as a first-order separable autoregressive process in two dimensions, such that the SAC between two values of a trait is modeled as a power function of the spatial distance between the values, in the x and y directions (Cullis and Gleeson 1991; Gilmour et al. 1997), and (2) incorporating home range overlap effects, by fitting an “S matrix” to the “animal model.” We examine the extent to which these effects explain variation and the effects of their inclusion on the key quantitative genetic parameters, *V*_A_ and *h*^2^, for each trait.

## Methods

### STUDY POPULATION AND DATA COLLECTION

The data in this study are taken from a wild population of red deer, *Cervus elaphus*, on the North Block of the Isle of Rum, Scotland, which has been intensively monitored since 1971. All individuals in the population can be recognized by artificial markings or by natural idiosyncrasies (Clutton-Brock et al. 1982). The study population was released from a culling regime in 1973, and the population size then rose steadily toward carrying capacity in the mid-1980s, with the current population fluctuating around approximately 200 adult females (Coulson et al. 2004). Females in this population associate in loosely matrilineal groups (Albon et al. 1992; Clutton-Brock et al. 1982). In contrast to females, young males disperse from their natal groups at around the age of two years (Clutton-Brock et al. 1982). Males born to the study population often return to the study area to rut, but outside of this essentially all adult males live outside the study area in other parts of the island for the majority of the year. Relatively little spatial information is therefore available across the lifetimes of males, and here we focus our analyses only on females.

**Figure 1:**
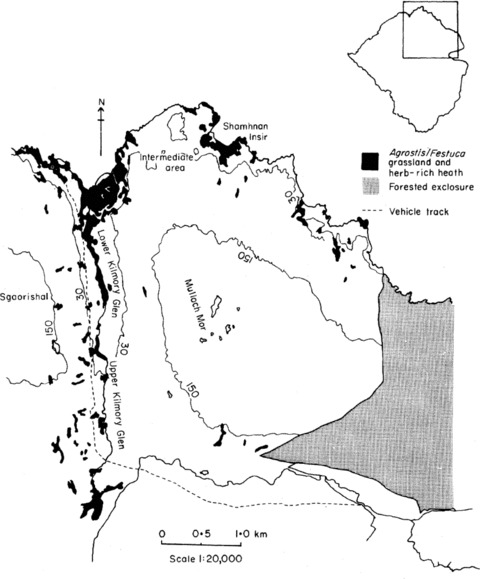
The study area, showing the distribution of *Agrostis/ Festuca* grassland (adapted from Guinness et al. 1978).

The study area is approximately 13 km^2^, comprising a gently sloping hill (Mulloch Mor) and the surrounding glens, with the majority (more than 70%) of the area lying below 120 m (Clutton-Brock et al. 1982). The north boundary of the study area follows 3.5 km of coastline from Kilmory Bay to another bay, Shamhnan Insir to the East (Fig. 1; Guinness et al. 1978). Females spend most of their time feeding along this coastal strip and around the North end of the Kilmory River, which runs down Kilmory Glen and drains into the bay (Clutton-Brock et al. 1982; Coulson et al. 2004; McLoughlin et al. 2006). Five main types of vegetation have been classified in the study area: *Agrostis/Festuca* grassland, *Juncus-*dominated marshland, *Molinia*-dominated flush, *Calluna-*dominated heath and heather moorland, and small patches of *Eriophorum*-dominated bog (Clutton-Brock et al. 1982; McLoughlin et al. 2006). There is considerable heterogeneity of these vegetation types across the study area and the use of areas rich in *Agrostis/Festuca* has been positively associated with female lifetime reproductive success in this population (McLoughlin et al. 2006).

Regular censusing of the study population throughout the year provides detailed spatial information and, coupled with regular mortality searches of the area, comprehensive records of both calf and adult mortality. In addition, during the calving season (May–June), detailed observations are taken of heavily pregnant females to identify when and where calves are born. This allows the majority (64% over the whole study period) of individuals born into the population to be caught shortly after birth, when they are sexed, weighed, and tissue sampled for genetic paternity analysis (see below). Capture weight is adjusted for the time since birth to give an estimated BW for each individual in kilograms (birth weight = capture weight –[0.01539 × age at capture in hours], following Clutton-Brock et al. 1982). Note that throughout our analyses, we treat BW as a trait of the mother, rather than of the offspring itself (e.g., Nussey et al. 2005b; Moyes et al. 2011). We also used breeding records to calculate lifetime breeding success as the number of offspring a female produced over her lifetime.

Locations of individuals during spring were taken from censuses conducted five times a month during the period of January–May. During a census, a fixed route is walked through the study area and the identity of all individuals seen is recorded and their grid reference noted to the nearest 100 m. Although censuses are undertaken in other months of the year, the data used here were restricted to that period because at other times individual location may be temporarily affected by calving or mating behaviors (Coulson et al. 1997). During the rut (15 September to 15 November), censuses are undertaken each day, again recording identity and location of individuals to the nearest 100 m (Stopher et al. 2011).

We investigated the effects of incorporating SAC and home range overlap on the estimation of quantitative genetic parameters in four female traits: spring home range size (SHR), rut home range size (RHR), offspring BW, and lifetime breeding success (LBS) (see Table 1 for a full list of abbreviations used in the manuscript). Note that although multiple measures per female across different years are available for the first three variables, LBS is measured only once. Previous studies have suggested that age, reproductive status, local population density, and region of the study area may be important determinants of these traits (Coulson et al. 2003; Moyes 2007; Nussey et al. 2009). Age is known for females and reproductive status has previously been categorized into five categories, as follows (see Clutton-Brock et al. 1982): “milk” (calved, and calf survived to at least 1 May the year after birth), “winter yeld” (calved, but calf died in the winter after birth, between 1 October and 1 May), “summer yeld” (calved, and calf died before 1 October), “true yeld” (the female did not calve the previous year), and “naïve” (the female had never previously calved). Females are considered to range mainly within one of five regions in the study area: Kilmory, Shamhnan Insir, Intermediate area, Mid glen, or South glen (Moyes 2007). Based on a female's mean annual location in spring or the rut, we assigned her to one of these regions, and then calculated local population size in this study for each region annually as the number of adult females whose mean location falls within each region.

**Table 1:** Abbreviations used in the manuscript

BA	Bhattacharyya's affinity
BW	Birth weight
*h*^2^	Heritability
LBS	Lifetime breeding success
LMM	Linear mixed effects model
RHR	Rut home range size
SAC	Spatial autocorrelation
SHR	Spring home range size
UD	Utilization distribution
UDOI	Utilization distribution overlap index
*V*_A_	Additive genetic variance
*V_I_*	Variance attributable to “*I*” (any additional random effect)
*V*_M_	Maternal variance
*V*_PE_	Permanent environment variance

### HOME RANGE ANALYSIS

**Figure 2:**
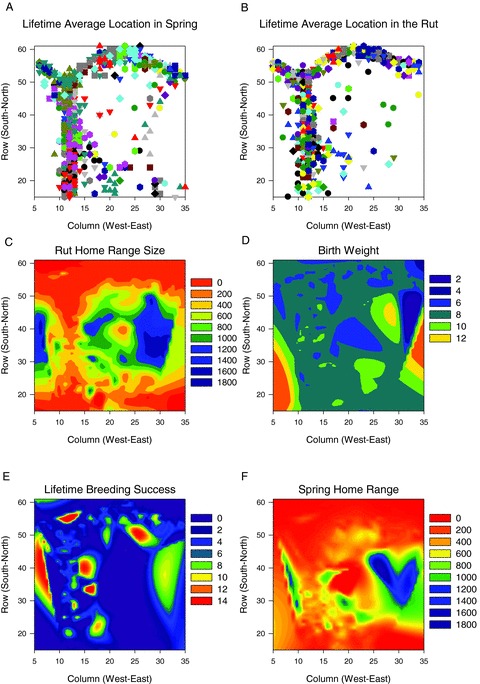
Spatial distributions of female red deer and traits analyzed across the Kilmory study area. (A and B) show the distribution of average female lifetime locations from spring censuses (Jan–May) and daily censuses from the rut (Sept–Nov), respectively (colors and symbols refer to matrilines originating from females alive at the start of the study). (C–F) show spatial distributions of different traits—rut home range size, spring home range size, offspring birth weight, and lifetime breeding success—using mean values for each 100-m grid square with females allocated to grid squares based on average lifetime locations. Where data are not available for a grid square, the expected value for that square is interpolated from those around it (using default algorithms implemented in SigmaPlot, Systat software 2008).

For the purposes of spatial analyses, the locations in which individuals were recorded were transformed on to a grid, so that the most south-westerly location recorded (135100, 798500) became (0, 0) and each step along the grid in either direction represented a shift in location by 100 m. Positions on the grid were then represented by a grid reference (column, row). Average lifetime locations of individuals on this grid are plotted in Fig. 2A (average location during January–May) and Fig. 2B (average location during the rut). Lifetime average locations were then used to account for SAC in animal models (see below).

Home range sizes were estimated for each female annually, separately, from home ranges estimated using locations recorded within spring and the rut. Core home ranges were estimated with kernel density estimation methods (Worton 1987, 1989; Borger et al. 2006) using the package “adehabitat” (version 1.8.3, Calenge 2006) in R version 2.8.1 (R Development Core Team 2008). Where fewer than 10 locations were recorded for an individual during a particular season, the data were excluded for that female. Previous work has shown that this number was sufficient for accurate home range estimation using techniques similar to those used here (Borger et al. 2006). However, we also tested whether the number of fixes used to estimate a home range influenced range size and accounted for the number of fixes as a fixed effect in models where this was the case. Finally, because censuses record the grid references of individuals to the nearest 100 m, many fixes have exactly the same grid reference. This can cause problems in the calculation of home range sizes and overlap using kernel methods (Tufto et al. 1996). To address this, we “jittered” locations used for home range estimation by adding a random number between –20 and 20 to the X and Y coordinates for each grid reference (following Moyes 2007).

Having generated home ranges and estimated home range size for all individuals, we went on to estimate the extent of home range sharing among individuals. To do this, we calculated home ranges as above, but using all locations recorded over an individual's lifetime rather than annual locations. Home range overlap was then calculated with Bhattacharyya's affinity (BA; Bhattacharyya 1943; Fieberg and Kochanny 2005). By using BA, individuals have an overlap of 1 with themselves; scaling from 0 to 1 in this way makes scaling of the overlap term comparable to that of relatedness between two individuals. This is essential when comparing the variance in a trait explained by the relatedness and spatial matrix because the variance explained by each matrix must be on the same scale. Full details of how home ranges and home range overlap were estimated are given in File 1 in supporting information.

We calculated a home range overlap matrix (S matrix) for all individuals in the genetic pedigree; where no home range information was available for an individual, it was assigned a home range overlap index of 1 with itself (diagonals set to 1), and was assumed to have an overlap of zero with all other individuals (missing off-diagonals assumed to be zero). Compared to 4051 individuals (1384 females) in the pedigree, home range information only existed for 948 females in spring and 766 females in the rut. However, the pedigree also contains missing information: 691 individuals have no known mother or father. Furthermore, any lack of information in the S matrix is likely to make our estimates of the variance in a trait explained by home range overlap conservative, and therefore any reduction in heritability on accounting for spatial similarity also conservative.

### PEDIGREE RECONSTRUCTION

All mothers are known through association with their calves, whereas genetic paternity analysis was used to assign fathers. As discussed above, the majority of individuals are caught at birth and samples taken for paternity analysis. Genetic sampling for individuals not caught at birth occurs from cast antlers, chemical immobilization, or postmortem. Prior to 1991, individuals were genotyped at up to eight highly variable microsatellites; since then individuals have been genotyped at up to 15 microsatellites. Detailed methods of pedigree construction are given in Walling et al. (2010). Two programs were used for paternity assignment: MasterBayes (Hadfield et al. 2006) and COLONY2 (Wang and Santure 2009). All assignments were made at greater than 80% individual confidence. Preference was given to paternity assignments made by MasterBayes, with COLONY2 assignments accepted where MasterBayes could not assign a father with greater than 80% confidence (following Walling et al. 2010).

### INCORPORATING SPATIAL INFORMATION INTO LINEAR MIXED MODELS

Linear mixed-effects models (LMMs) were conducted in ASReml3 (VSN International, Hemel Hempsted, UK; Gilmore et al. 2009). We used two techniques to incorporate spatial information into the models. First, we fitted average lifetime spatial coordinates as ordered row and column effects, fitted as additional random effects, with a covariance structure that assumed a first-order separable autoregressive process to account for spatial dependence (AR1 × AR1, Gilmour et al. 1997). Second, we incorporated information on home range overlap between individuals into the animal model by fitting a vector of shared home range effects as an additional random effect, with the corresponding covariance matrix 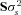, where **S** is the home range overlap matrix (the “S matrix”). For full details of how we incorporated spatial information into linear mixed models, please see File 2 in supporting information.

### MODEL FITTING AND SIGNIFICANCE TESTING

Significance of random effects was assessed using likelihood ratio tests and fixed effects were assessed using Wald statistics. We built models of four traits (RHR, SHR, BW, and LBS) in three stages: (1) testing of fixed effects deemed likely to be of importance based on previous research and retaining those terms that were significant; (2) incorporating random effects to measure additive genetic, permanent environment, maternal effects, and annual variance, and then (3) incorporating additional random effects to model SAC or home range overlap. We then examined the magnitude and significance of these spatial effects and the effects of including them on the magnitude of other random effects, particularly the additive genetic variance component, in our models. It was necessary to log-transform RHR, SHR, and LBS prior to analysis in order that the distribution of the residuals had a closer approximation to normality.

Fixed effects for female age (linear and quadratic terms) and reproductive status were tested for all traits, and sex of offspring was tested for BW. The number of fixes used to calculate an annual home range was included in models of RHR and SHR. Fixed effects related to spatial processes were also tested, namely region of the study area and local population size. These potentially account for some of the spatial heterogeneity in these traits. However, it has been argued that although fitting such trends is unlikely to change estimates of quantitative genetic parameters, their inclusion can aid our understanding of the nature of the spatial variation present, and improve the likelihood of achieving stationarity in models incorporating SAC (Dutkowski et al. 2002). An illustration of the broad-scale spatial distribution of the four traits is presented in Fig. 2C–F. The following fixed effects were found to be significant in models of each trait, and were included in subsequent LMMs: RHR: age, region, local population size, count of fixes used to calculate home range size; SHR: age, age^2^, local population size, region, reproductive status (note: count of fixes was not significant); BW: age, age^2^, reproductive status, region, and offspring sex; LBS: region.

We added random effects sequentially. First, we included a random effect for individual identity to estimate among individual variation. Second, a term modeling the variance attributable to phenotypic similarity among relatives was included using relatedness information from the pedigree in an “animal model.” This model separates among-individual variation into additive genetic (*V*_A_) and so-called “permanent environment” (*V*_PE_) components. We subsequently included a random effect of year of measurement and the identity of the individual's mother, to estimate variation attributable to variation in annual environment (*V*_Year_) and maternal effect (*V*_M_) both of which have been shown to be important in previous studies of this population (Kruuk et al. 2000; Kruuk and Hadfield 2007). Note that, because LBS was only measured once per individual, only *V*_A_, *V*_M_, and *V*_Year_ were included for this trait and year of birth had to be used rather than year of measurement.

We then incorporated either SAC or S matrix into these base LMMs as described above. Note that if the data structure allows, the two could be incorporated simultaneously into one model. When including SAC, we estimated both the correlation parameter and the variance in the trait explained by the spatial term on fitting (1) a column process, (2) row processes, and (3) column and row processes.

It is important to note that, while adding SAC to animal models revealed interesting environmental sources of variance (see Results), some models did not produce credible results. In particular, some estimates of the variance explained by spatial processes were extremely large (see Table 2, e.g., SHR models with row processes included). Model credibility was checked by summing the variance components estimated by each model. Although some minor changes in the variance explained are not necessarily a particular concern, changes in the order of magnitude of the total variance are rather alarming and suggest the model has produced a poor estimate of the variance components. This occurred particularly when the estimates of the SAC were bounded at 1 (i.e., could not be estimated). As shown in Table 2, this did not seem to be an issue in models including both row and column processes except for SHR, for which only models incorporating column processes produced reasonable variance estimates. When discussing SAC models below, we therefore focus on models incorporating row and column process for RHR, BW, and LBS but only column processes for SHR. Such difficulties were not evident when fitting the S matrix.

**Table 2:** Variance components from models including no spatial effect, spatial autocorrelation (either column or row process or both), or a matrix of home range overlap (S matrix) for four traits in wild red deer. For each of the five models presented, “Var.” is the variance component for each random effect and “Prop.” is the proportion of the total variance in the random effects model explained by that term (with standard errors in brackets). Italicized variance components are those that were bound at 0 or 1. “Sum V” is the sum of variance components; substantial changes in this value as spatial terms are added are assumed to reflect poor estimation of components in the model

	No spatial effect		Column		Row		Column and row		S matrix	
					
	Var.	Prop.	Var.	Prop.	Var.	Prop.	Var.	Prop.	Var.	Prop.
Rut home range										
*V*_PE_	*0.000 (0.000)*	*0.000 (0.000)*	*0.000 (0.000)*	*0.000 (0.000)*	*0.000 (0.000)*	*0.000 (0.000)*	*0.000 (0.000)*	*0.000 (0.000)*	*0.000 (0.000)*	*0.000 (0.000)*
*V*_A_	0.168 (0.020)	0.314 (0.032)	0.110 (0.015)	0.022 (0.008)	0.104 (0.014)	0.078 (0.129)	0.043 (0.009)	0.035 (0.012)	0.001 (0.003)	0.001 (0.003)
*V*_Year_	0.017 (0.005)	0.031 (0.009)	0.016 (0.005)	0.003 (0.002)	0.016 (0.005)	0.012 (0.020)	0.016 (0.005)	0.013 (0.005)	0.013 (0.004)	0.015 (0.005)
*V*_M_	0.084 (0.018)	0.158 (0.031)	0.067 (0.015)	0.013 (0.006)	0.037 (0.010)	0.028 (0.046)	0.024 (0.007)	0.020 (0.009)	0.006 (0.003)	0.007 (0.004)
*V*_Column_			4.550 (1.704)	0.909 (0.031)						
*V*_Row_					0.917 (2.237)	0.687 (0.519)				
*V*_Columnandrow_							0.893 (0.383)	0.723 (0.086)		
*V*_Smatrix_									0.598 (0.120)	0.681 (0.045)
*V*_Residual_	0.265 (0.006)	0.497 (0.021)	0.265 (0.006)	0.053 (0.018)	0.263 (0.006)	0.197 (0.326)	0.260 (0.006)	0.211 (0.066)	0.260 (0.006)	0.296 (0.041)
Column ϕ			*0.991 (0.000)*				0.970 (0.014)			
Row ϕ					0.959 (0.103)		0.935 (0.028)			
Sum *V*	0.534		5.008		1.337		1.236		0.878	
*h*^2^ (%)	31.31		2.196		7.779		3.479		0.114	
Spring home range										
*V*_PE_	*0.000 (0.000)*	*0.000 (0.000)*	*0.000 (0.000)*	*0.000 (0.000)*	*0.000 (0.000)*	*0.000 (0.000)*	*0.000 (0.000)*	*0.000 (0.000)*	*0.000 (0.000)*	*0.000 (0.000)*
*V*_A_	0.193 (0.017)	0.437 (0.028)	0.119 (0.012)	0.210 (0.032)	0.108 (0.011)	0.011 (0.004)	0.044 (0.006)	0.003 (0.001)	0.002 (0.002)	0.003 (0.003)
*V*_Year_	0.012 (0.004)	0.026 (0.008)	0.010 (0.003)	0.018 (0.006)	0.010 (0.003)	0.001 (0.000)	0.009 (0.003)	0.001 (0.000)	0.008 (0.002)	0.012 (0.004)
*V*_M_	0.017 (0.009)	0.039 (0.020)	0.016 (0.007)	0.029 (0.013)	0.002 (0.005)	0.000 (0.001)	0.005 (0.004)	0.000 (0.000)	0.000 (0.002)	0.000 (0.002)
*V*_Column_			0.201 (0.066)	0.355 (0.076)						
*V*_Row_					9.723 (3.167)	0.966 (0.011)				
*V*_Columnandrow_							13.511 (3.603)	0.980 (0.005)		
*V*_Smatrix_									0.487 (0.088)	0.693 (0.039)
*V*_Residual_	0.220 (0.005)	0.498 (0.019)	0.219 (0.005)	0.388 (0.046)	0.217 (0.005)	0.022 (0.007)	0.215 (0.005)	0.016 (0.004)	0.206 (0.004)	0.293 (0.037)
Column ϕ			0.410 (0.177)				0.987 (0.005)			
Row ϕ					*0.999 (0.000)*		*0.999 (0.000)*			
Sum *V*	0.442		0.565		10.060		13.784		0.703	
*h*^2^ (%)	43.666		21.06		1.074		0.319		0.284	
Birth weight										
*V*_PE_	0.049 (0.068)	0.033 (0.047)	0.050 (0.069)	0.033 (0.047)	0.112 (0.067)	0.009 (0.008)	0.109 (1.610)	0.063 (0.043)	0.082 (0.000)	0.054 (0.046)
*V*_A_	0.530 (0.098)	0.356 (0.055)	0.517 (0.099)	0.347 (0.057)	0.362 (0.087)	0.029 (0.020)	0.364 (4.160)	0.212 (0.076)	0.402 (0.041)	0.268 (0.055)
*V*_Year_	0.081 (0.025)	0.054 (0.016)	0.081 (0.025)	0.054 (0.016)	0.081 (0.025)	0.007 (0.005)	0.049 (1.150)	0.047 (0.019)	0.107 (0.067)	0.071 (0.016)

	No spatial effect		Column		Row		Column and row		S matrix	
					
	Var.	Prop.	Var.	Prop.	Var.	Prop.	Var.	Prop.	Var.	Prop.
*V*_M_	0.046 (0.043)	0.031 (0.029)	0.049 (0.043)	0.033 (0.029)	0.049 (0.043)	0.004 (0.004)	0.081 (3.270)	0.029 (0.026)	0.038 (0.088)	0.025 (0.027)
*V*_Column_			0.008 (0.017)	0.006 (0.012)						
*V*_Row_					10.986 (7.525)	0.888 (0.069)				
*V*_Columnandrow_							0.336 (0.700)	0.195 (0.224)		
*V*_Smatrix_									0.088 (0.074)	0.059 (0.048)
*V*_Residual_	0.783 (0.029)	0.526 (0.027)	0.783 (0.029)	0.527 (0.028)	0.781 (0.029)	0.063 (0.039)	0.781 (0.029)	0.454 (0.128)	0.782 (0.029)	0.529 (0.036)
Column ϕ			0.491 (0.330)				*0.999 (0.000)*			
Row ϕ					*0.999 (0.000)*		0.967 (17.510)			
Sum *V*	1.489		1.488		12.371		1.720		1.499	
*h*^2^ (%)	35.594		34.745		2.926		21.162		26.818	
Lifetime breeding success										
*V*_A_	0.009 (0.008)	0.044 (0.039)	0.007 (0.007)	0.038 (0.038)	0.011 (0.008)	0.011 (0.011)	0.008 (0.007)	0.041 (0.037)	0.000 (0.000)	0.000 (0.000)
*V*_Year_	0.077 (0.020)	0.394 (0.064)	0.075 (0.020)	0.385 (0.064)	0.076 (0.020)	0.078 (0.061)	0.076 (0.020)	0.386 (0.064)	0.040 (0.011)	0.245 (0.053)
*V*_M_	0.007 (0.005)	0.038 (0.026)	0.007 (0.005)	0.035 (0.026)	0.008 (0.005)	0.008 (0.008)	0.007 (0.005)	0.034 (0.025)	0.003 (0.003)	0.016 (0.021)
*V*_Column_			0.003 (0.002)	0.015 (0.012)						
*V*_Row_					0.789 (0.730)	0.803 (0.147)				
*V*_Columnandrow_							0.005 (0.004)	0.027 (0.020)		
*V*_Smatrix_									0.045 (0.010)	0.275 (0.052)
*V*_Residual_	0.103 (0.009)	0.525 (0.071)	0.102 (0.009)	0.528 (0.071)	0.099 (0.009)	0.100 (0.076)	0.100 (0.009)	0.512 (0.070)	0.076 (0.006)	0.465 (0.052)
Column ϕ			–0.775 (0.225)				–0.894 (0.129)			
Row ϕ					*0.999 (0.000)*		0.951 (0.074)			
Sum *V*	0.196		0.194		0.983		0.196		0.164	
*h*^2^ (%)	4.592		3.608		0.011		4.082		0.000	

*V*_A_, additive genetic effect; *V*_PE_, permanent environment effect; *V*_Year_, annual environment effect; *V*_M_, maternal effect; *V*_Column_, variance attributable to column spatial processes; *V*_Row_, variance attributable to row spatial processes; *V*_Columnandrow_, variance attributable to both column and row spatial processes; *V*_Smatrix_, variance attributable to home range overlap matrix; *V*_Residual_, residual variance; Column ϕ, spatial autocorrelation estimate for column processes; Row ϕ, spatial autocorrelation estimate for row processes; Sum *V*, sum of variance component in model.

## Results

**Figure 3:**
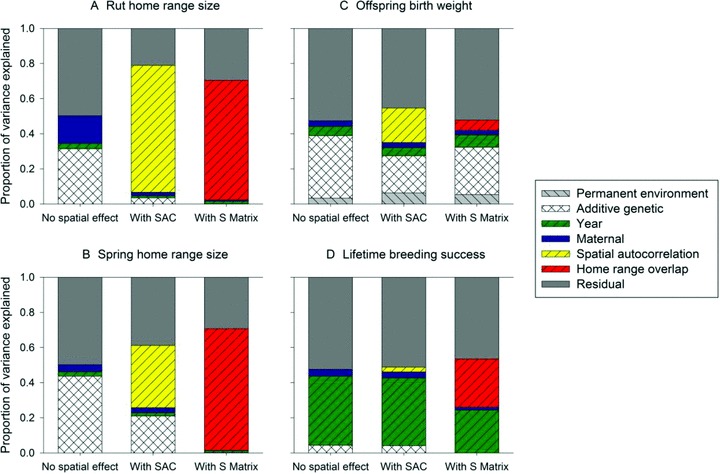
The proportion of variance in four different traits explained by different random effects in models including no spatial effects, spatial autocorrelation terms (“with SAC,” column and row processes, except for birth weight where only column processes were included as parameter estimates appeared poorly estimated when row processes were included, see Table 2), or a home range overlap (or “S”) matrix (with S matrix).

Fixed effects coefficients for each trait are given in File 3 in supporting information. In models without spatial processes, RHR was moderately heritable with a negligible permanent environment effect and small but significant maternal and year effects (Table 2; Fig. 3A). Model fit was significantly improved when SAC was added (inclusion of column and row processes: χ^2^_(df = 3)_ = 369.9, *P* < 0.001), and the SAC coefficients reveal strong positive autocorrelation along both column (west-east) and row (south-north) axes (Table 2). SAC explained 72% of the variance. Inclusion of SAC resulted in a substantial reduction of the estimated *V*_A_ and *V*_M_ and also in the proportion of the total variance explained by these effects (heritability from 31% to 3%, maternal effect from 8% to 2%), as well as reductions in the year and residual terms (Table 2; Fig. 3A). Inclusion of the “S matrix” also significantly improved model fit (χ^2^_(1)_= 785.8, *P* < 0.001) compared to fitting no spatial information, and this term explained substantial variance in RHR (68%; Fig. 3A). Its inclusion resulted in reductions of even greater magnitude in *V*_A_ and *V*_M_ than seen in the SAC model, with heritability becoming negligible (Table 2; Fig. 3A).

SHR was highly heritable and, like RHR, showed negligible *V*_PE_ along with small *V*_M_ and *V*_Year_ components (Table 2; Fig. 3B). SAC effects were highly significant (inclusion of column processes: χ^2^_(df = 2)_= 179.7, *P* < 0.001) and explained 36% of the variance (Fig. 3B). As for RHR, there was evidently strong positive SAC and incorporating this into the model resulted in large reductions in estimated *V*_A_ and heritability (from 44% to 21%; Table 2; Fig. 3B). Inclusion of the “S matrix” also significantly improved model fit compared to fitting no spatial information (χ^2^_(df = 1)_= 1313.2, *P* < 0.001). Home range overlap explained 69% of the variance and resulted in dramatic declines in all other variance components, with heritability dropping to <1% (Table 2; Fig. 3B).

BW was moderately heritable, with only small amounts of variance attributable to *V*_PE_, *V*_M_, and *V*_Year_ (Table 2; Fig. 3C). Although region was a significant fixed effect in BW models, its inclusion resulted in singularities when we attempted to include SAC processes in models, presumably because the two are heavily confounded. Exclusion of region had little effect on the estimation of other variance components and we therefore present BW models without region as a fixed effect for comparison in Table 2 and Fig. 3C. Generally, BW models including SAC were quite unstable (e.g., see the large standard errors of spatial variance components and the frequency with which spatial processes were bounded at 1); they should be interpreted with caution. However, inclusion of SAC did significantly improve model fit (inclusion of column and row processes: χ^2^_(3)_= 20.5, *P* < 0.001), and column and row processes explained around 20% of the variance in BW (Table 2; Fig. 3C). Positive SAC coefficients suggested that females living in close proximity have similar offspring birth weights, although the column process estimate was bound at one (Table 2). In models including SAC effects, estimates of *V*_A_ were reduced and heritability declined from 36% to 21% (Fig. 3C). Addition of the S matrix term to a model of birth weight including region as a fixed effect improved model fit compared to fitting no spatial information (χ^2^_(1)_= 5.3, *P* < 0.05) but it explained only 6% of the variation. The estimated heritability of BW in a model including region was 28.2% (± 5.66 SE) and inclusion of the S matrix term resulted in only a small reduction in heritability (to 25.6 ± 5.54%).

LBS was weakly heritable, with a small maternal effect and substantial cohort variation (Table 2; Fig. 3D). Addition of SAC resulted in a marginally nonsignificant improvement in model fit (inclusion of column and row processes: χ^2^_(3)_= 7.58, *P*= 0.056) and explained less than 3% of the variance, and this came mostly from the residual variance with very little change in heritability (Table 2; Fig. 3D). Interestingly, although the spatial variance was small, the estimated SAC parameters trended toward positive SAC of LBS in the south-north direction (row) but negative autocorrelation in the west-east direction (column; Table 2). Finally, there was a highly significant home range overlap effect on LBS (χ^2^_(1)_= 185.6, *P* < 0.001), compared to fitting no spatial effects, with home range overlap explaining 28% of the variance in LBS (Fig. 3D). Inclusion of the S matrix in the model resulted in a decrease in all other variance components, with estimated heritability becoming negligible and the cohort effect declining from 39% to 25% (Table 2; Fig. 3D).

For RHR, SHR, and LBS, comparison of AICs showed that models including the S matrix outperformed models with SAC processes fitted (comparing model with S matrix to model with row and column processes: RHR: –1958.10 vs. −1126.32, SHR: −4875.16 vs. −3407.07, LBS: −1875.57 vs. −1519.54). However, for BW, the AICs of the two models were similar, with the model including SAC processes having slightly lower AIC: 4263.16 versus 4274.96.

## Discussion

Our analyses show that evolutionary biologists and ecologists working in natural systems should consider modeling fine-scale spatial processes if they want to fully understand the environmental drivers of phenotypic variation and accurately estimate quantitative genetic parameters. Accounting for shared environmental effects associated with either SAC or home range overlap, over and above effects of maternal identity, cohort and region of the study area, resulted in decreases in *h*^2^ of up to an order of magnitude (e.g., RHR, Table 2). Furthermore, both SAC and S matrix approaches provided new insight into the way spatial heterogeneity in resources influences key behavioral, life-history, and fitness traits. Interestingly, both the variance explained by SAC or the S matrix and their effects on *h*^2^ estimates varied markedly depending on the trait in question (Table 2; Fig. 3). Furthermore, the variance explained by SAC was greater than that explained by the S matrix in some traits (e.g., RHR, BW) but the opposite was true for others (e.g., SHR, LBS).

To our knowledge, only one study previous to ours has addressed the effects of SAC between trait values in related individuals in a wild animal population (van der Jeugd and McCleery 2002). That study suggested SAC resulted in overestimation of heritability of lay date in the great tit (although not clutch size), suggesting our findings are not specific to this study system. The extent of the effect of SAC on other traits and species remains however to be seen. In any system where there is incomplete or nonrandom dispersal of relatives and the habitat is heterogeneous, relatives are more likely to experience the same environment than would be expected by chance and this shared environmental experience will result in phenotypic resemblance that does not have a genetic basis (unless there is a genetic component to habitat choice itself, see below). However, the extent to which this biases estimates of heritability will vary with the amount to which related and nonrelated individuals are distributed within an environment, the extent to which the environment varies over the studied area, and the extent to which environmental and genetic factors determine trait values. Below, we discuss possible reasons for the differences we have found between female red deer traits in the effect of SAC on heritability estimates. We also consider the relative merits of the SAC and S matrix approaches, and highlight the potential for developing and implementing fitting additional covariance matrices within evolutionary ecology.

### DIFFERENCES IN SPATIAL EFFECTS AMONG TRAITS

To our knowledge, this is the first study to estimate the heritability of home range size in a wild mammal. Quantitative genetic studies of traits associated with dispersal, ranging, and foraging behavior remain rare in wild mammals (e.g., Waser and Jones 1989), although they are the focus of increasing interest in birds (e.g., Doligez et al. 2009; Charmantier et al. 2011; Teplitsky et al. 2011). Although initial models suggested high *V*_A_ and *h*^2^ in both RHR and SHR in red deer, and a moderate maternal effect in RHR, these effects all but disappear once either SAC or home range overlap have been accounted for (Fig. 1A, B). This starkly illustrates the potential pitfalls of failing to account for space or habitat sharing in an “animal model.” In both home range traits, substantial proportions of total variance were attributable to positive spatial autocorrelation or home range sharing, indicating that individuals with average lifetime locations in close proximity, or those that shared large proportions of their lifetime home range, had similar home range sizes. This is not surprising: home range size is likely to be closely associated with food availability, with individuals having to range further to meet energetic demands if they live in poor quality habitats (McNab 1963). Forage availability and quality varies markedly across our study area, and our results are likely to reflect increased home range sizes and reduced home range overlap among females living in regions of poorer vegetation in the south and east of the North Block (McLoughlin et al. 2006; Moyes 2007).

The importance of spatial effects on both BW and LBS were smaller than for home range sizes and estimates of *h*^2^ were accordingly less biased by their exclusion. Quantitative genetic estimates from the models accord well with previous studies: our BW estimates of *V*_A_ and *V*_PE_ are similar to those for maternal genetic and environmental variance from a study that treated this as an offspring trait (Kruuk and Hadfield 2007), although estimates for LBS were slightly higher than previous work that found zero heritability (Kruuk et al. 2000). The latter difference could be attributable to our larger present dataset, an improved pedigree, or the inclusion of a cohort random effect in our models. For both BW and LBS, we found that a substantial proportion of variance (around 20% and 30%, respectively) was attributable to either SAC or home range overlap. This suggests fine-scale spatial effects are important for life-history and fitness-correlated traits as well as those associated with ranging behavior. Previous work has identified significant spatial heterogeneity in fitness linked to the relationships between use of *Agrostis/Festuca* grassland, local population density, and lifetime reproductive success, and suggested this heterogeneity could be maintained by social constraints to dispersal preventing females from moving to more productive areas (McLoughlin et al. 2006, 2008). Although the mechanisms linking spatial location or home range overlap with BW and LBS variation remain to be determined, our results illustrate how estimation of SAC or S matrix effects could be used to provide insight into their relative importance for demographic variation and population dynamics in wild animals.

The contrasting relative importance of SAC versus home range overlap effects in some traits suggests differences in the processes linking resource heterogeneity and phenotype. For example, although both SAC and S matrix accounted for comparably large proportions of variation in RHR, the S matrix explained considerably more variation in SHR (Fig. 3A, B). SAC models of SHR were notably unstable (Table 2), so the difference here could be due an inability of the model to estimate the variance explained by SAC. However, there are biological reasons to expect differences: resource availability increases over the spring period but declines over the autumn, and female home ranges shrink substantially during the rut and may fall under some degree of influence of male rutting behavior (Clutton-Brock et al. 1982, although see Stopher et al. 2011). Interestingly, SAC but not home range overlap explained variation in BW but the reverse was true for LBS. Why spatial location per se rather than home range overlap should explain variance in BW is unclear; it could reflect the importance of the specific area a female tends to use during the gestation and lactation periods. This is supported by the fact that models including region as a fixed effect would not converge, and suggests a wider scale of resource variation may be important. The relative importance of home range sharing, rather than spatial location, for LBS variation may reflect fine-scale constraints associated with local competition in high-density and resource quality regions in the north of the study area, where home ranges are likely to overlap extensively (McLoughlin et al. 2006, 2008). There is tentative support for this in the SAC models that show nonsignificant negative autocorrelation in the column (east-west direction), but positive SAC in the row (south-north) direction (Table 2). In ecological studies, negative SAC is indicative of competition, such that individuals with high trait values depress the trait values of neighbors (Dutkowski et al. Dukowtski 2002, Haining 2004). The distribution of females in the study area means that the majority of column process information comes from the North, moving east from Kilmory to Shamhnan Insir (Figs. 1,2), where high local densities would be expected to drive greater competition for resources.

### DEVELOPING THE MULTIMATRIX APPROACH IN EVOLUTIONARY ECOLOGY

Our results suggest that exploring SAC and home range overlap effects side-by-side could be biologically informative, and other studies may also wish to explore the wider range of statistical methods developed for accounting for SAC (see for example Dutkowski et al. 2002). However, we would argue that fitting the matrix of home range overlap is the more appropriate way to deal with causes of environmental similarity between relatives. This is because patterns of space use, as indicated by home range overlap, are more likely to accurately describe the similarity of the environment two individuals experience, in terms of available food and shelter, and the energy they have to expend to acquire these. Because we used a home range overlap index that included information on the utilization distribution of home ranges (i.e., the amount individuals actually use different parts of the home range), our S matrix gives a very accurate measure of extent to which individuals experience similar environmental conditions. In contrast, using an average location is a cruder measure of the environment an individual experiences, not least because the error on the estimate of average location is likely to vary between individuals, depending upon the differences in the extent to which animals range around that average location. A comparison of model AICs shows that models including home range overlap performed better than models including SAC processes for three of four traits. Further, we found that models including SAC were not necessarily stable in the parameters they estimated, or in their likelihood of converging. In contrast, models using the double-matrix approach were straightforward to fit and converged. These considerations imply that, faced with a choice, ecologists and evolutionary biologists should favor the use of home range overlap or resource-sharing matrices rather than SAC functions.

It is striking that we found such strong effects of home range overlap on the traits considered despite the existence of certain limitations in our S matrix approach. For example, the matrix uses lifetime home ranges, and includes no information about when individuals existed: it therefore assumes individuals with identical home ranges separated by as much as 30 years experience the same environmental conditions. Ideally therefore, temporal information on overlap of individuals in time as well as space would be incorporated, or the matrix could be constructed on an annual basis. However, producing home range overlap matrices for large populations is not trivial and incorporating temporal variation in these matrices into animal models is not going to be straightforward.

Further, it is important to exercise caution when interpreting the results of this, or any similar study, to not assume that estimated heritabilities are free from bias even if shared environment effects are accounted for. For example, if there exists a genetic component to habitat choice, such that individuals choose habitats according to their genotypes, variance apparently explained by shared environmental effects may have an underlying genetic component. Accounting for shared environment effects may therefore result in underestimation of genetic variance. In this study, this may not be a problem, as females do not disperse and therefore have little opportunity to “choose” an environment, but were there a genetic component to the location of home range such a bias could exist, and future studies using such techniques should be aware of the issue. In general, as we begin to think about ways to more fully account for environmental similarity between relatives, it will be important to question whether additive genetic variance is to some extent absorbed by the environmental term and therefore downwardly biased. In this study, the pedigree, although imperfect, is more complete than the fitted S matrix, implying that this is unlikely. However, it may be a problem for other systems, particularly where the pedigree is shallow. In light of these limitations, future studies (including simulation studies) that examine how home range overlap matrices and other environmental similarity matrices could be best computed, the factors that affect the ability to separate genetic and environmental variance using such models, and what additional biological insight they could bring, would certainly be worthwhile in light of our results.

In general, this “double matrix” technique—fitting both genetic relatedness and environmental similarity—offers exciting possibilities for separating the causes of similarity between individuals. Fitting additional covariance matrices is a common practice in animal breeding to dissect different genetic contributions to phenotypic variation (e.g., additive, dominance, and epistatic effects: e.g., Smith and Maki-Tanila 1990; Palucci et al. 2007). A recent review has strongly advocated the separation of transmissible nongenetic effects using additional matrices capturing shared resources or social interactions (Danchin et al. 2011). To our knowledge, ours is the first study to empirically implement such an approach and it clearly highlights both the potential for confounding effects of fine-scale shared environmental effects on *V*_A_ and *h*^2^, as well as the ecological importance of such effects on phenotypic variation.

Beyond spatial analysis, additional covariance matrices could be fitted to animal models to assess the variance explained in traits by association between individuals. The use of social network analysis has recently become very popular in behavioral ecology to identify and quantify the interactions between individuals and the extent to which individuals associate (Wey et al. 2008). The approach has been used to describe social structure and predict patterns of cooperation in guppies (Croft et al. 2004, 2006), and spatial-association networks in bats are thought to be important in not just in social life but also in epidemiology (Rhodes et al. 2006; Wey et al. 2008). Furthermore, the fitness correlates of social relationships are not well known (but see Silk et al. 2003, 2010). Methods to incorporate social association information into quantitative genetic analysis are currently an area of much endeavor (see Walsh and Lynch 2009). However, a recent study stated that matrices of genetic relatedness and social interactions could not be fitted simultaneously within an “animal model” (Frere et al. 2010), yet our study shows that this should be perfectly possible, given a data structure that allows the separation of genetic and social variance, by fitting a matrix of interactions between individuals, that is, an association matrix (Whitehead 2008), to an “animal model” of a fitness trait. Should sufficient data be available, with sufficient independence between the matrices to allow their separation, this could potentially even be extended to a model in which similarity between individuals in wild populations was separated into relatedness, shared environment, and social associations.

## Supporting Information

The following supporting information is available for this article:

**Table S1.** Coefficients and standard errors of fixed effects for each trait.
