# Supplementary material for: SHARED SPATIAL EFFECTS ON QUANTITATIVE GENETIC PARAMETERS: ACCOUNTING FOR SPATIAL AUTOCORRELATION AND HOME RANGE OVERLAP REDUCES ESTIMATES OF HERITABILITY IN WILD RED DEER
Source: Evolution. 2012 Aug;66(8):2411–26. doi: 10.1111/j.1558-5646.2012.01620.x (PMC3437482; doi:10.1111/j.1558-5646.2012.01620.x)
Supplement: Supplementary file 1 [file evo0066-2411-SD1.pdf]

Supplementary File 3: Fixed Effects Cofficents

Table 1. Coefficients and standard errors of fixed effects for each trait. Values are taken from a model with only the reported variables fitted as fixed effects and additive genetic (V_A) , permanent environment ($V_{PE,}$ not for LBS) year of measurement (V_{Year}, cohort for LBS) and the identity of the individual's mother (V_M) fitted as random effects. See Methods and Results for more detail.

Fixed Effect	Level of effect	Coefficient	Standard Error
**RHR**			
No. of fixes		-0.020	0.001
Local population size		-0.006	0.001
Region	Kilmory	0.000	0.000
Region	South Glen	1.089	0.075
Region	Mid Glen	0.719	0.078
Region	Intermediate	0.162	0.073
Region	Shamhnan Insir	-0.848	0.077
Age		-0.007	0.003
**SHR**			
Reproductive status	Naïve	0.000	0.000
Reproductive status	Winter Yeld	-0.187	0.036
Reproductive status	True Yeld	-0.004	0.034
Reproductive status	Summer Yeld	0.002	0.037
Reproductive status	Milk	-0.081	0.031
Region	South Glen	0.000	0.000
Region	Shamhnan Insir	-1.283	0.059
Region	Mid Glen	-0.445	0.037
Region	Kilmory	-1.082	0.059
Region	Intermediate	-0.847	0.058
Local population size		-0.004	0.001
Age2		-0.003	0.000
Age		0.035	0.009
**BW**			
Sex	Female	0.000	0.000
Sex	Male	0.344	0.044
Reproductive status	Naïve	0.000	0.000
Reproductive status	True Yeld	0.547	0.093
Reproductive status	Winter Yeld	-0.054	0.107
Reproductive status	Summer Yeld	0.547	0.097
Reproductive status	Milk	0.129	0.094
MumRegion	Intermediate	0.000	0.000
MumRegion	South Glen	0.631	0.152
MumRegion	Kilmory	0.105	0.127
MumRegion	Shamhnan Insir	-0.102	0.125
MumRegion	Mid Glen	0.467	0.152
Age		-0.018	0.002
Age2		0.336	0.045

**LBS**			
Region	South Glen	0	0
Region	Mid Glen	0.1847	0.5272
Region	Intermediate	0.1016	0.4342
Region	Shamhnan Insir	-0.242	0.4167
Region	Kilmory	0.4727	0.4015
