# Supplementary material for: SHARED SPATIAL EFFECTS ON QUANTITATIVE GENETIC PARAMETERS: ACCOUNTING FOR SPATIAL AUTOCORRELATION AND HOME RANGE OVERLAP REDUCES ESTIMATES OF HERITABILITY IN WILD RED DEER
Source: Evolution. 2012 Aug;66(8):2411–26. doi: 10.1111/j.1558-5646.2012.01620.x (PMC3437482; doi:10.1111/j.1558-5646.2012.01620.x)
Supplement: Supplementary file 2 [file evo0066-2411-SD2.pdf]

Supplementary File 1: full details of home range and home range overlap estimation methods.

Home ranges were estimated using kernel density estimation methods (Borger et al. 2006; Worton 1987; Worton 1989). This type of home range estimation has revolutionised the concept of an animal's home range, as it takes into account the utilization distribution of the animal, that is, the probability distribution defining the animal's use of space (Fieberg and Kochanny 2005; Kenward et al. 2001; Van Winkle 1975; Worton 1989). It has been recommended that multiple smoothing parameters are used and the results compared (Borger et al. 2006). We attempted to model home range sizes using both the reference bandwidth, h_{ref}, method and the least squares cross validation method. However, the latter method performed poorly and would not converge, a finding which has been predicted by other studies for home ranges with relatively small numbers of fixes (Seaman and Powell 1996). We therefore used the h_{ref} method. We estimated home ranges using the package 'adehabitat' (version 1.8.3, Calenge 2006) in R version 2.8.1 (R Development Core Team 2008).

We estimated home ranges for each individual in each season at a very wide range of isopleths (between 20 and 95% at 5% intervals), where isopleths indicate the probability of finding an individual within the calculated home range. If home ranges are used randomly, then a positive linear correlation exists between isopleth and home range size; however, if animals use part of the home range more intensely then a plot of home range size against isopleth will be curved beneath the line of random use. At the point at which the difference between random use and actual use is greatest, the isopleth represents the 'core home range' of the animal (Powell 2000). In our analysis, the core home range was found to be that with a 70% isopleth for both spring and rut home ranges. The size of the 70% isopleth home range

in hectares is therefore presented as ‘spring home range size’ (SHR) and ‘rut home range size’ (RHR) hereafter.

Having generated home ranges and estimated home range size for all individuals, we went on to estimate the extent of home range sharing among individuals. To do this, we used home ranges calculated as above, but using all locations recorded over an individual’s lifetime rather than annual locations. Although home range overlap can be calculated as a simple proportion of an animal *i*'s home range that is overlapped by an animal *j*’s home range (Kernohan et al. 2001), incorporating information about an animal’s utilization distribution (UD) can result in more informative measures of home range overlap. Not doing so can result in a large overlap estimate even though the probability of finding two individuals in the same place is small (Fieberg and Kochanny 2005). Fieberg and Kochanny (2005) recommend the use of either the UD overlap index (UDOI) or Bhattacharyya’s affinity (BA; Bhattacharyya 1943) as measures for quantifying home range overlap on this basis. UDOI is arguably the most appropriate measure of shared space use, because it can take into account the degree to which utilization distributions are concentrated in space, whereas BA is more appropriate to quantify the overall similarity between UDs. However, BA has the advantage that it ranges from zero to 1, equalling one when UDs are uniformly distributed with 100% overlap. Here, we used BA and we calculated home range overlap at a 100% isopleths, rather than a (core) 70% isopleth. By using BA calculating home range overlap at 100%, individuals have an overlap of 1 with themselves; scaling from 0-1 in this way makes scaling of the overlap term comparable to that of relatedness between two individuals. This would not be the case if using the UDOI method. This is essential when comparing the variance in a trait explained by the relatedness and spatial matrix because the variance explained by each matrix must be on the same scale.

References:

Bhattacharyya, A. 1943. On a measure of divergence between two statistical populations defined by their probability distributions. Bulletin of the Calcutta Mathematical Society 35: 99-109.

Borger, L., Franconi, N., De Michele, G., Gantz, A., Meschi, F., Manica, A., Lovari, S. & Coulson, T. 2006. Effects of sampling regime on the mean and variance of home range size estimates. Journal of Animal Ecology 75: 1393-1405.

Calenge, C. 2006. The package "adehabitat" for the R software: A tool for the analysis of space and habitat use by animals. Ecological Modelling 197: 516-519.

Fieberg, J. & Kochanny, C. O. 2005. Quantifying home-range overlap: The importance of the utilization distribution. Journal of Wildlife Management 69: 1346-1359.

Kenward, R. E., Clarke, R. T., Hodder, K. H. & Walls, S. S. 2001. Density and linkage estimators of home range: Nearest-neighbor clustering defines multinuclear cores. Ecology 82: 1905-1920.

Kernohan, B. J., Gitzen, R. A. & Millspaugh, J. J. (2001) Anaysis of animal space use and movements. In: Radio tracking animal populations, (Millspaugh, J. J. & Marzluff, J. M., eds.). pp. Academic Press, San Diego, California.

Powell, R. A. (2000) Animal home ranges and territories and home range estimators. In: Research Techniques in Animal Ecology Controversies and Consequences, (Boitani, L. & Fuller, T. K., eds.). pp. Columbia University Press, New York.

Seaman, D. E. & Powell, R. A. 1996. An evaluation of the accuracy of kernel density estimators for home range analysis. Ecology 77: 2075-2085.

Van Winkle, W. 1975. Comparison of Several Probabilistic Home Range Models. Journal of Wildlife Management 39: 118-123.

Worton, B. J. 1989. Kernel Methods for Estimating the Utilization Distribution in Home-Range Studies. Ecology 70: 164-168.
