# Supplementary material for: SHARED SPATIAL EFFECTS ON QUANTITATIVE GENETIC PARAMETERS: ACCOUNTING FOR SPATIAL AUTOCORRELATION AND HOME RANGE OVERLAP REDUCES ESTIMATES OF HERITABILITY IN WILD RED DEER
Source: Evolution. 2012 Aug;66(8):2411–26. doi: 10.1111/j.1558-5646.2012.01620.x (PMC3437482; doi:10.1111/j.1558-5646.2012.01620.x)
Supplement: Supplementary file 3 [file evo0066-2411-SD3.pdf]

Supplementary File 2: Incorporating spatial information into linear mixed models

A general mixed model written in matrix form is given by:

$$y = \boldsymbol{X}\beta + \boldsymbol{Z}u + e$$

where y is the vector of data, β is a vector of fixed effects with design matrix $\boldsymbol{X}$, u is a vector of random effects with design matrix $\boldsymbol{Z}$ and e is a vector of residual errors (Eisenhart 1947, Lynch and Walsh 1998). For a simple animal model containing only an additive genetic term and residual error, this is therefore:

$$y = \mu + a + e$$

Where $\boldsymbol{X}$ is now a vector of ones, β has become μ, $\boldsymbol{Z}$ is the identity matrix and u is now $a,$ the vector of additive genetic effects. In the animal model, $\boldsymbol{G}$ is defined as sum of the variance-covariance matrices of each of the random effects. In this example, $\boldsymbol{G}$ is therefore given by $\boldsymbol{A}\sigma^2{}_A$ where $\boldsymbol{A}$ is the additive genetic relationship matrix (Kruuk 2004). $\boldsymbol{R}$ is defined as the variance-covariance matrix of the residuals (Kruuk 2004). If the residuals are assumed to be independent, $\boldsymbol{R}$ is defined as $\boldsymbol{I}\sigma^2{}_e$ where $\boldsymbol{I}$ is the identity matrix. However, spatial autocorrelation between the residuals can be accounted for by splitting e into spatially dependent (ξ) and spatially independent residuals (η). Using a covariance structure that assumes a 1st order separable autoregressive process to account for spatial dependence (AR1xAR1, Gilmour et al. 1997), the autocorrelation is a power function of the distance apart so that in two dimensions separable autoregressive processes for columns and rows are

modelled such that r($X_{i,j}$, $X_{k,l}$) = $\rho row^{|i-k|}\rho col^{|j-l|}$ for individuals with row (i,j) and column (k,l) coordinates (Cullis and Gleeson 1991). Doing so, $\boldsymbol{R}$ therefore becomes:

$\boldsymbol{R} = \sigma_{\xi}^2[AR1(\rho_{col})\otimes AR1(\rho_{row})] + \sigma_{\eta}^2\boldsymbol{I}$ (Dutkowski *et al.* 2002)

In this study, we chose to define an equivalent linear model in which the same covariance structure was applied to ordered row and column effects and included in $\boldsymbol{G}$ rather than $\boldsymbol{R}$. This allows the use of an incomplete spatial array, but is otherwise equivalent (Dutkowski *et al.* 2002, Apiolaza 2006).

As an alternative approach to incorporating spatial information, we also fitted the 'S matrix' of home range overlap information, in which case the model in matrix form becomes:

$$y = \mu + a + s + e$$

where s is the vector of shared home range effects, with the corresponding covariance matrix $\boldsymbol{S}\sigma^2{}_S$, where $\boldsymbol{S}$ is the home range overlap matrix, with elements:

$$\boldsymbol{S_{ij}} = \iint_{-\infty-\infty}^{\infty\infty} \sqrt{\widehat{UD}_i(\boldsymbol{x},\boldsymbol{y})} \times \sqrt{\widehat{UD}_j(\boldsymbol{x},\boldsymbol{y})\ \boldsymbol{dx}\,\boldsymbol{dy}}$$

where $\widehat{UD}_j(\boldsymbol{x},\boldsymbol{y})$ is the estimated value of the utilization distribution of the animal j at the point $(\boldsymbol{x},\boldsymbol{y})$ **(**Feiberg and Kochanny 2005).

References

Apiolaza, L. A. (2006) ASReml cookbook. pp. http://uncronopio.org/ASReml/.

Cullis, B. R. & Gleeson, A. C. 1991. Spatial-Analysis of Field Experiments - an Extension to 2 Dimensions. Biometrics 47: 1449-1460.

Dutkowski, G. W., Silva, J. C. E., Gilmour, A. R. & Lopez, G. A. 2002. Spatial analysis methods for forest genetic trials. Canadian Journal of Forest Research-Revue Canadienne De Recherche Forestiere 32: 2201-2214.

Eisenhart, C. 1947. The assumptions underlying the analysis of variance. Biometrics 3: 1-21.

Fieberg, J. & Kochanny, C. O. 2005. Quantifying home-range overlap: The importance of the utilization distribution. Journal of Wildlife Management 69: 1346-1359.

Gilmour, A. R., Cullis B.R. & Verbyla, A.P. 1997. Accounting for natural extraneous variaion in the analysis of field experiments. JABES 2: 269-273.

Kruuk, L. E. B. 2004. Estimating genetic parameters in natural populations using the 'animal model'. Philosophical Transactions of the Royal Society of London Series B-Biological Sciences 359: 873-890.

Lynch, M. W., B. 1998. Genetics and analysis of quantitative traits. Sinauer Associates, Inc.
